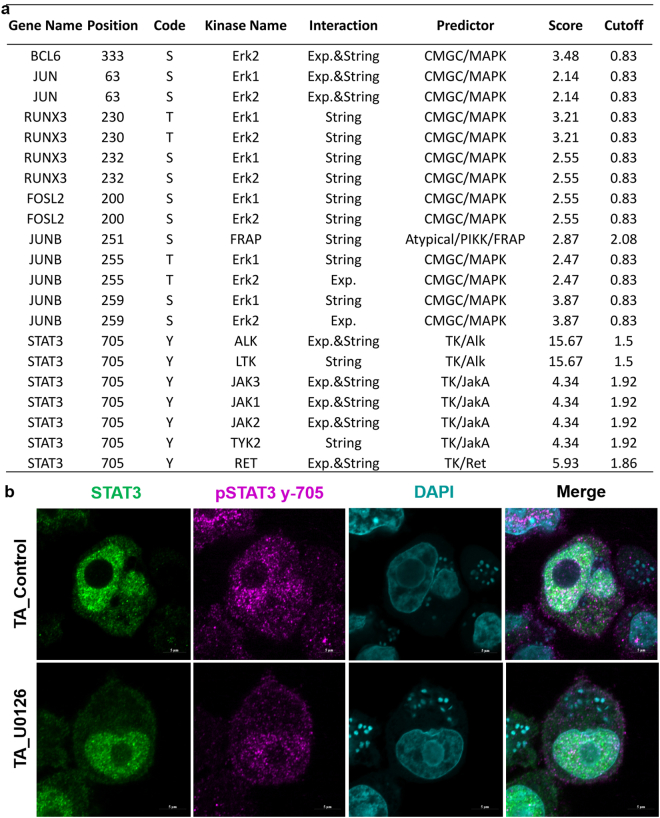# *Theileria annulata* Hijacks Host Signaling: Integrated Phosphoproteomics and Transcriptomics Unveils ERK1/2 as a Central Regulator of Host Transcription Factors

**DOI:** 10.1016/j.mcpro.2025.100992

**Published:** 2025-05-12

**Authors:** Debabrata Dandasena, Vengatachala Moorthy A, Akash Suresh, Vasundhra Bhandari, Sonti Roy, Paresh Sharma

**Affiliations:** 1 Host Microbe Interactions and Epigenetics Laboratory, National Institute of Animal Biotechnology, Hyderabad, Telangana, India; 2 Department of Biological Sciences (Pharmacoinformatics), National Institute of Pharmaceutical Education and Research (NIPER), Hyderabad, Telangana, India

**Keywords:** cancer-like transformation, kinase-mediated signaling, phosphoproteomics, *Theileria annulata*, transcriptomics, transcription factor regulation

## Abstract

*Theileria*-transformed bovine leukocytes exhibit cancer-like characteristics, but the molecular mechanisms driving these transformations remain unclear. This study provides the first comprehensive phosphoproteomic analysis of both host and parasite in *Theileria annulata*–infected leukocyte cell lines. We show that *T. annulata* significantly induces changes in the host protein phosphorylation, impacting key cancer-related processes such as apoptosis suppression, CAMK signaling, and telomere maintenance. A pivotal finding is the parasite’s manipulation of the MAPK pathway via sustained ERK1/2 activation, which regulates the phosphorylation of critical transcription factors like RUNX3, FOSL2, BCL6, c-JUN, JUNB, and c-MYC. Transcriptomic analysis of genes controlled by these transcription factors confirmed their role in *T. annulata* replication. ERK inhibition disrupts phosphorylation, deactivates these transcription factors, and induces apoptosis in infected cells. This underscores the ERK-AP-1 axis as a central mechanism of *Theileria* pathogenesis and a promising therapeutic target. Additionally, parasite-specific phosphoproteins and kinases were identified, offering new insights into therapeutic strategies to combat infection.

## Highlights

• *Theileria annulata* infection triggers dynamic changes in host cell phosphorylation with ERK1/2 emerging as a central regulator.
• Parasite clearance disrupts these phosphorylation events, highlighting their importance for *T. annulata* survival.
• ERK1/2 signaling influences the expression and/or phosphorylation of specific transcription factors, potentially impacting the host cell response.
• Disrupting ERK1/2 signaling impairs parasite proliferation, reduces parasite burden, and triggers host cell apoptosis, suggesting a potential therapeutic target.
• Unveils a novel mechanism by which *T. annulata* manipulates host cell signaling for its own benefit.

## Graphical Abstract

**Figure:**
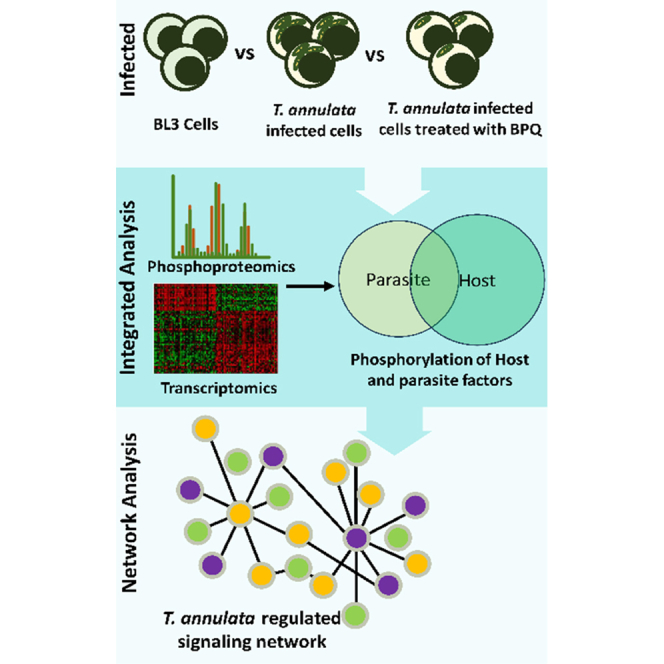

## In Brief

This study reveals how *Theileria annulata* manipulates host signaling pathways by inducing widespread phosphorylation changes, particularly through sustained ERK1/2 activation, which regulates key transcription factors (RUNX3, FOSL2, BCL6, c-JUN, JUNB, c-MYC) to promote parasite survival. Integrated phosphoproteomics and transcriptomics demonstrate that ERK1/2 inhibition disrupts this axis, triggering apoptosis in infected cells. The findings highlight the ERK-AP-1 pathway as central to *Theileria* pathogenesis and identify potential therapeutic targets, including parasite-specific kinases, for combating infection.

*Theileria annulata*, a tick-borne apicomplexan parasite, is the causative agent of tropical theileriosis, a disease of significant economic burden to the cattle industry across Asia, Africa, and Southern Europe (1). This tick-borne disease results in substantial production losses, highlighting the need for improved control strategies. The parasite targets lymphocytes, crucial components of the adaptive immune system, leading to a spectrum of debilitating clinical manifestations, including pyrexia (fever), anemia, and lymphadenopathy. Despite its substantial economic and agricultural impact, our understanding of the intricate molecular mechanisms employed by *T. annulata* to manipulate host cell signaling cascades and establish a successful infection remains incomplete. While some studies have explored these mechanisms (2, 3), a comprehensive understanding is still lacking. This knowledge gap hinders the development of effective control strategies for tropical theileriosis. Developing effective strategies to combat *T. annulata* infection necessitates a deeper understanding of the intricate molecular mechanisms underlying parasite–host interactions.

Infection of bovine leukocytes by *T. annulata* parasites triggers a profound reprogramming of host cell signaling cascades (4). This manipulation results in a transformed phenotype, characterized by dysregulated proliferation and the acquisition of replicative immortality (1, 5). Intriguingly, a viable parasite is essential for both transformed cell survival and phenotype, indicating these changes are driven by manipulated signaling and gene expression (5). *Theileria* infection disrupts host leukocyte signaling, evidenced by altered kinase activity and protein phosphorylation (6). Protein phosphorylation is a fundamental regulatory mechanism in eukaryotic cells, governing a wide range of cellular processes (7). Kinases, a class of enzymes responsible for attaching phosphate groups to proteins, act as key regulators of signaling cascades that govern cell growth, survival, and differentiation. *Theileria* parasites orchestrate intricate posttranslational modifications (PTMs) within their host cells to manipulate essential processes and establish successful infections. For instance, constitutive NF-κB activation safeguards *Theileria*-infected cells from apoptosis (8). Similarly, AP-1–driven MMP9 expression facilitates *Theileria*-infected leukocyte dissemination (9).

While the complete PTM landscape remains undiscovered, phosphorylation events mediated by kinases are recognized as crucial regulators. Previous studies have shown that *Theileria*-transformed cells activate a diverse array of kinases to sustain transformed cell survival, including mitogen-activated protein kinases (MAPKs), c-Jun N-terminal kinase (JNK), SRC family kinases, casein kinase-2 (CK2), and phosphatidylinositide 3-kinase and polo-like kinase 1 (3, 6, 10, 11, 12, 13, 14, 15, 16). Parasite proteins like p104 (sequestering JNK2) and secreted TaPIN1 (stabilizing c-Jun) have been shown to contribute to this manipulation (13, 17). However, the exact mechanisms triggering such activations in these parasitized cells remain largely unknown. This coordinated activation of multiple signaling pathways by the parasite likely represents a mechanism to manipulate host cell cycle progression and survival, highlighting the need for further investigation. These findings highlight the importance of kinases in *Theileria*'s life cycle. However, a comprehensive understanding of the host and the parasite's kinomes and its influence on PTM profiles during infection requires further exploration using advanced phosphoproteomic techniques. Unraveling the parasite's kinome and its impact on PTMs holds significant promise for identifying novel therapeutic targets and developing effective control strategies for *Theileria*-induced infections. Traditional approaches for studying protein phosphorylation often rely on the investigation of specific kinases or known signaling pathways. However, these methods may miss crucial, yet unidentified, phosphorylation events. Global phosphoproteomics, a powerful technique that comprehensively analyzes and quantifies protein phosphorylation across the entire cellular proteome, offers an unbiased approach to uncover the global kinase-mediated signaling landscape during *T. annulata* infection.

In this study, we leverage the power of global phosphoproteomics and transcriptomics to investigate the kinome landscape in bovine lymphocytes infected with *T. annulata*. We employ high-throughput mass spectrometry to identify and quantify phosphopeptides, providing a comprehensive view of protein phosphorylation events triggered by the parasite. By comparing the phosphoproteome profile of infected cells to uninfected controls, we aim to identify differentially phosphorylated proteins and associated kinases. Our investigation identified novel phosphorylation sites within host and parasite proteins mediated by *T. annulata*. We have also demonstrated the intricate regulation of multiple transcription factors (TFs) by kinases. Furthermore, RNA-seq analysis of hallmarks associated with cancer reveal the profound influence of these TFs on gene expression. These findings underscore the critical role of these factors in promoting the observed proliferative phenotype in *T. annulata*–transformed cells. Deciphering the complex interplay within host cell signaling pathways elicited by *T. annulata* lays the groundwork for the discovery of novel therapeutic targets, with the potential to revolutionize our approach to combating this pathogen.

## Experimental procedures

### Experimental Design and Statistical Rationale

This study involved three experimental conditions: uninfected control BL3 cells, *T. annulata*–infected cells (TA), and *T. annulata*–infected cells treated with the parasite-specific drug buparvaquone (TA_BPQ) to clear the parasite. Two biological replicates were analyzed per condition, with each replicate corresponding to four technical replicates, resulting in a total of 12 data files. Phosphopeptides with a localization score exceeding 0.75 were retained to ensure high confidence in phosphorylation site identification. Phosphoprotein data was filtered to include only those with valid values in at least 50% of the experimental groups. Missing data points were imputed using a combination of normal distribution and K-Nearest Neighbor methods. ANOVA was employed to assess statistical significance, using a predefined false discovery rate (FDR) threshold of 0.05, with 250 randomizations performed to account for potential chance associations. Z-score normalization was applied to standardize the data and enable meaningful comparisons between the experimental groups. These filtering criteria were chosen as a compromise between statistical stringency and the number of data points retained after processing. Statistical analyses ensured robust identification of differentially phosphorylated proteins across the three conditions.

### *In vitro* Cell Culture

Bovine peripheral blood mononuclear cells were isolated from cattle diagnosed with clinical *T. annulata* infection. Following isolation, peripheral blood mononuclear cells were cultured in RPMI 1640 medium supplemented with 10% fetal bovine serum (FBS) and 100 μg/ml penicillin-streptomycin solution. These cultures were maintained under controlled conditions at 37 °C in a humidified incubator with 5% CO2 (adapted from 37). PCR amplification was performed to ensure the successful establishment of *T. annulata* infection within the cultured bovine cells. Primers specific to the *T. annulata*–specific TaSP gene were utilized (18). A positive PCR result confirmed the presence of *T. annulata* parasites within the *in vitro* cell culture system. The BL3.1 bovine B lymphosarcoma cell line, obtained from ATCC, was maintained in RPMI 1640 medium supplemented with 10% FBS. This cell line served as a control for subsequent experiments. To investigate the effects of specific drugs on *Theileria*-infected cells, treatments were administered under the following conditions: buparvaquone at 200 ng/ml and U0126 at 10 μM. The duration of drug exposure varied depending on the experiment and is specified within the individual results sections.

### Workflow for Proteomics Sample Preparation and LC-MS/MS Analysis

**Supplemental Figure 1:**
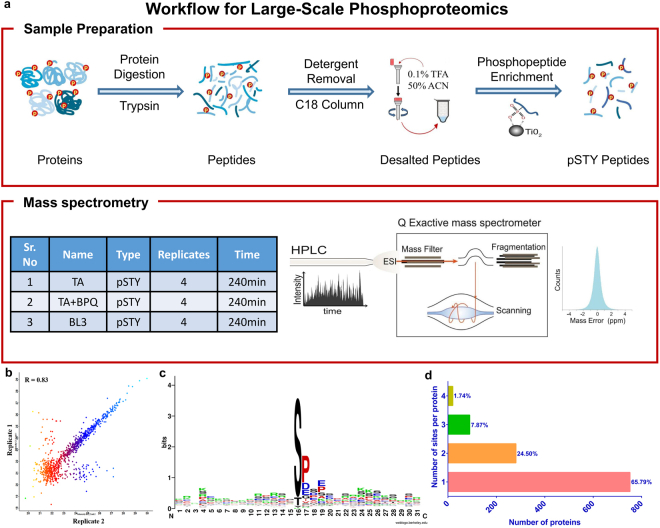
Workflow for Phosphoproteomics.

Following specific treatment durations, samples from control and treated cells were collected and lysed with reagents prepared in ammonium bicarbonate water with protease/phosphatase inhibitors. Samples underwent washes, sonication, centrifugation, and protein quantification (BCA). Proteins were then digested with trypsin (1:50 w/w) for 16 h at 37 °C. Digested peptides were desalted using C18 columns and enriched for phosphopeptides using a TiO2 kit (supplemental Fig. S1*A*). Enriched phosphopeptides (500 ng) were separated on an Easy Spray nano LC C18 column (Thermo Fisher Scientific),75 μm d. x 500 mm. at a flow rate of 200 nl/min on an Ultimate 3000 nano LC system (Thermo Fisher Scientific) by altering the gradient: 0%–5% B (80% acetonitrile with 0.1% formic acid) in 10 min, 5%–40% B in 210 min, 40%–70% B in 15 min, 70%–90% in 10 min, 90%–5% B in 4 min, 5%–0% B in 1 min. 240-min gradient was performed for whole proteome and phosphoproteome analyses. An Orbitrap Q Exactive HF instrument (Thermo Fisher Scientific) was operated in the data-independent mode with a full scan in the Orbitrap. For phosphoproteome analyses, full scans were performed with a resolution of 60,000, a target value of 1 × 10^6^ ions, and a maximum injection time of 60 ms. The data-independent acquisition (DIA) scans were performed with 30,000 resolution, a 1 × 10^6^ target value, and a 60 ms maximum injection time. Isolation window was set to 40 m/z and normalized collision energy was 27. The MS1 data was acquired with m/z ranges from 385 to 1015.

### LC-MS Data Analysis for Phosphopeptide Identification

MaxQuant software (version 2.0.3.0) facilitated the analysis and processing of raw mass spectrometry data (19). Search parameters allowed for a maximum of two missed cleavages during trypsin digestion and considered the fixed modification of cysteine carbamidomethylation. Additionally, for phosphoproteome analysis, variable modifications encompassing phosphorylation of serine, threonine, and tyrosine residues were incorporated. The search parameters included a peptide mass tolerance of 6 ppm for MS scans and 20 ppm for MS/MS scans. The “match between runs” feature was enabled to enhance data quality during analysis. The Andromeda search engine within MaxQuant was employed against a combined database containing the UniProt *Bos taurus* proteome (UP000009136_9913.fasta) and *T. annulata* proteome (UP000001950), along with common contaminant entries. A stringent FDR of 1% was applied at both the peptide and protein levels for reliable identification. Protein quantification required a minimum of two unique peptide identifications (“ratio counts”), and the “match between runs” setting was activated for improved data consistency. Following successful protein identification, Perseus software (versions 1.6.15.0 & 2.0.9.0) was utilized to statistically analyze the identified peptides and their corresponding proteins to pinpoint significantly altered phosphoproteins (20). The downstream analysis considered both the abundance (intensity) of phosphopeptides and the confidence score for the localization of modified amino acid residues. Only phosphopeptides with a localization score exceeding 0.75 were retained for further analysis, ensuring high confidence in phosphorylation site identification. Additional statistical parameters included the following: i) Data filtering: phosphoprotein data was filtered to ensure the presence of valid values in at least half (50%) of the experimental groups; ii) Imputation of missing values: missing data points were imputed using a combination of normal distribution and K-Nearest Neighbor methods; iii) Multiple testing correction: statistical significance was assessed using ANOVA with a predefined FDR of 0.05. This analysis involved randomization repeated 250 times to account for potential chance associations and iv) Data normalization: Z-score normalization was employed to standardize the data and facilitate statistical comparisons between different experimental groups.

### *In silico* Prediction of Kinase–Substrate Interactions

The software and resources used to predict kinase–substrate relationships between parasite and host proteins were as follows:

#### Parasite Kinase Prediction

The publicly available group-based prediction system (GPS) 6.0 server (https://gps.biocuckoo.cn/online.php) was utilized to analyze the protein sequences of *T. annulata* kinases for potential phosphorylation sites.

#### Host Kinase Prediction

The standalone version of group-based prediction system with the interaction filter (iGPS) v1.0.1 software was used to analyze the complete host proteome for potential kinase phosphorylation sites.

#### Kinome Tree Visualization

The CORAL online tool (http://phanstiel-lab.med.unc.edu/CORAL/) (21) was employed to generate a visual representation of the kinases (kinome tree).

#### Prediction and Weighting Strategy

Both iGPS and GPS predicted potential kinase phosphorylation sites within protein sequences. A weighted scoring system was implemented to prioritize kinases based on the prediction frequency. For each protein sequence, a weight was assigned to each predicted kinase using the following formula: Weight = 1/(Number of predictions for that specific site and protein). This approach assigns a higher score to kinases that are frequently predicted to target a particular phosphorylation site. Subsequently, the cumulative weights for each kinase across all predictions were calculated.

#### Kinome Tree Representation

The cumulative kinase weights were then used to determine the relative size of circles within the kinome tree generated by CORAL. Kinases with a higher cumulative weight, indicative of a greater likelihood of targeting host proteins based on in silico predictions, were represented by larger circles within the tree.

### Gene Ontology Enrichment Analysis

#### Functional Annotation of Phosphorylated Sites

To functionally characterize the phosphorylation sites specific to *T. annulata* (TA) cells, we performed Gene Ontology (GO) enrichment analysis using the DAVID online tool (22). This analysis identified biological processes, molecular functions, and cellular components significantly enriched with these unique phosphorylation sites.

#### Visualization of KEGG Pathways

Following the GO enrichment analysis, we focused on the top five enriched KEGG pathways. These pathways were visualized to gain insights into the potential functional consequences of parasite-mediated phosphorylation events.

### Protein–Protein Interaction Network Analysis

Following the identification of transcription factors through phosphoproteomic analysis, we utilized the STRING 10 database (23) to explore potential protein–protein interactions. The analysis was performed with default settings in the confidence view to focus on high-confidence interactions. The resulting protein–protein interaction network was subsequently imported into Cytoscape software (version 3.0) for further analysis and visualization customization, as described in the Results section.

### Downstream Gene Analysis of Identified Transcription Factors

To identify potential downstream target genes regulated by the transcription factors identified through phosphoproteomics in *T. annulata* (TA)-infected bovine cells, we employed three resources: ENCODE, ChEA, and MotifMap4 databases. These databases catalog experimentally validated downstream genes associated with human TFs based on techniques like ChIP-seq experiments. The downstream target genes identified from the databases were compared to our RNA-seq data. We focused on diffrentially expressed genes exhibiting a fold change threshold of −1.5 (downregulated upon BPQ treatment) potentially indicative of TF activity. GO enrichment analysis was then performed using DAVID to identify biological processes and pathways potentially regulated by these TFs and their downstream targets.

### RNA sequencing

Control and treated bovine cells infected with parasites (BPQ at 200 ng/ml for 96 h) were lysed using TRIzol reagent, followed by phase separation with chloroform and centrifugation. The RNA-containing supernatant was purified using an RNeasy mini kit with on-column DNase I treatment. RNA concentration and purity were assessed using a Nanodrop spectrophotometer and Tapestation system. RNA concentration was further quantified using the Qubit RNA HS assay kit. Illumina-compatible RNA-seq libraries were prepared from 500 ng of total RNA using the NEBNext Ultra II Directional RNA Library Prep Kit according to the manufacturer's instructions. Briefly, the protocol involved mRNA isolation, fragmentation, priming, cDNA synthesis, purification, end-repair, adapter ligation, indexing PCR, and final library purification. Quality control steps included library quantification using a Qubit fluorometer and fragment size analysis using an Agilent TapeStation. Libraries were sequenced on a NovaSeq 6000 platform with 150 bp paired-end reads. Raw sequencing data underwent quality assessment with FastQC, followed by adapter sequence and low-quality base trimming using TrimGalore. Alignments were performed against the *B. taurus* (host) and *T. annulata* (pathogen) reference genomes using Hisat2 and Bowtie2, respectively. FeatureCounts tool was employed to quantify transcript abundance, and DESeq software was used to identify differentially expressed genes based on a log2 fold-change cut-off of ± 1. Differentially expressed genes were categorized as upregulated, downregulated, or neutrally regulated.

### RNA Extraction and Quantitative Real-Time PCR (qPCR)

Total RNA was isolated from the cells using the commercially available Quick-RNA Microprep Kit (Zymogen), adhering to the manufacturer's instructions. One microgram (1 μg) of the purified RNA was then reverse transcribed into complementary DNA (cDNA) using the PrimeScript first Strand cDNA Synthesis Kit (DSS Takara). Quantitative real-time PCR (qPCR) analysis was performed using the TB Green Premix Ex Taq II (RNaseH Plus) kit (DSS Takara), following the manufacturer's established protocol. This analysis quantified the relative expression levels of the genes. The housekeeping gene HPRT was employed for the normalization of the data. The specific primer sequences utilized for RT-qPCR are detailed: FOSL2 Forward GAGCCTTTCAAACCAGAGGGA FOSL2 reverse ATGGGCTGGACATGGAAGTG, HPRT For: TGGACAGGACCGAACGGCT and HPRT Rev: TAATCCAACAGGTCGGCAAG.

### Immunofluorescence Assay

Cells (5 × 10^5^) infected with the target agent were cultured for 48 h in media supplemented with either 10 μM U0126 or 200 ng/ml BPQ. A control group received no drug treatment. Following incubation, cells were harvested, washed with PBS (1X), and fixed with 4% paraformaldehyde for 15 min at 37 °C. Cells were then permeabilized with 0.1% Triton X-100 and blocked with 2% bovine serum albumin in PBS for 1 h at room temperature. Primary antibodies (detailed in the Experimental procedures section with specific dilutions) were incubated overnight at 4 °C. Unbound primary antibodies were removed by thorough washes with PBS containing 0.05% Tween-20. Subsequently, secondary antibody incubation (details in Experimental procedures section) was performed for 1 h at room temperature. Following further washes, cells were stained with DAPI for nuclear visualization. Finally, samples were mounted and imaged using a Zeiss Airyscan super-resolution microscope with a 100x objective. Image processing and quantification were performed using ZEN 3.3 software (Black Edition or Blue Edition) depending on the specific analysis requirements.

### SDS-PAGE and Western Blotting

Following treatment, cells cultured in six-well plates were washed with ice-cold PBS to remove residual media components. Cells were then lysed in radioimmunoprecipitation assay buffer containing protease and phosphatase inhibitors (compositions detailed in the Experimental procedures section) on ice for a designated time period (e.g., 15 min) to ensure complete protein extraction. Cell lysates were collected by scraping and clarified by centrifugation at high speed (e.g., 12,000*g*) at 4 °C for a specified duration (e.g., 15 min) to remove insoluble debris. Protein concentration in the clarified lysates was quantified using a bicinchoninic acid assay kit (detailed in the Experimental procedures section) according to the manufacturer's instructions. Lysate aliquots containing equal amounts of protein were then prepared for SDS-PAGE and Western blotting. Samples were prepared for SDS-PAGE by heat denaturation at 95 °C for 10 min in loading buffer containing reducing agents (e.g., β-mercaptoethanol) to disrupt protein disulfide bonds and ensure linear protein structure. Resolved proteins were then electrotransferred from the polyacrylamide gel to polyvinylidene difluoride membranes using a semidry or wet transfer system according to the manufacturer's protocol. Membranes were subsequently blocked with 5% (w/v) nonfat dry milk in Tris-buffered saline with 0.05% Tween-20 for 1 h at room temperature to minimize nonspecific antibody binding. Following blocking, membranes were incubated with primary antibodies (detailed in the Experimental procedures section with specific dilutions) overnight at 4 °C. Unbound primary antibodies were removed by thorough washes with Tris-buffered saline with 0.05% Tween-20. Membranes were then incubated with horseradish peroxidase-conjugated secondary antibodies (detailed in the Experimental procedures section) for a designated period at room temperature. Finally, protein bands were detected using enhanced chemiluminescent reagent and visualized using a digital imaging system.

#### siRNA Experiments

Commercially sourced siRNAs targeting the FOSL2 gene were acquired from Eurogentec. Two distinct siRNA sequences were utilized:

5′- (ACC-AGA-GGG-AUU-GAA-AUU-C)TT -3′ (siRNA_FOSL2_1)

5′- (ACU-GCA-GAA-AGA-GAA-AGA-G)TT -3′ (siRNA_FOSL2_2)

Lipofectamine 3000 transfection reagent (Invitrogen) facilitated the delivery of siRNAs into the cells, following the manufacturer's established protocol. Briefly, 3 × 10ˆ5 cells were seeded per well of a 6-well plate. A transfection cocktail was prepared by combining 5 μg of siRNA with 125 μl of serum-free RPMI medium and 7.5 μl of Lipofectamine 3000 in a separate aliquot of 125 μl serum-free RPMI medium. Following a 15-min incubation at room temperature, the combined solution was added to the cells in 1 ml of serum-free RPMI medium. After 6 h, the culture medium was supplemented with FBS to achieve a final concentration of 10%. Four days post-transfection, the cells were either utilized for subsequent downstream experiments or harvested for evaluation of knockdown efficiency via qPCR analysis.

### Statistical Analysis

Data are presented as either mean ± SEM for normally distributed data or median with interquartile range for non-normally distributed data. Normality of data was assessed using appropriate tests. Statistical comparisons were performed using unpaired t-tests for normally distributed data with equal variances, nonparametric Mann-Whitney U tests for non-normally distributed data or unequal variances, or one-way ANOVA for multiple comparisons. GraphPad Prism was used for all statistical analyses and generation of graphs. A *p*-value ≤0.05 was considered statistically significant unless otherwise specified. Specific details regarding the statistical tests used for each experiment are provided in the corresponding figure legends.

### Materials

#### Antibodies

**Figure:**
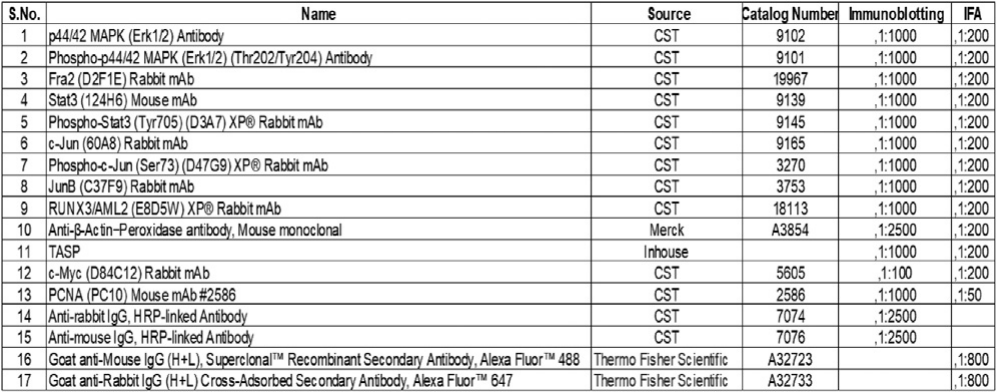

## Results

### Data-Independent Acquisition and Label-Free Quantification Reveal Dynamic Phosphorylation Events During *T. annulata* Infection

**Fig. 1:**
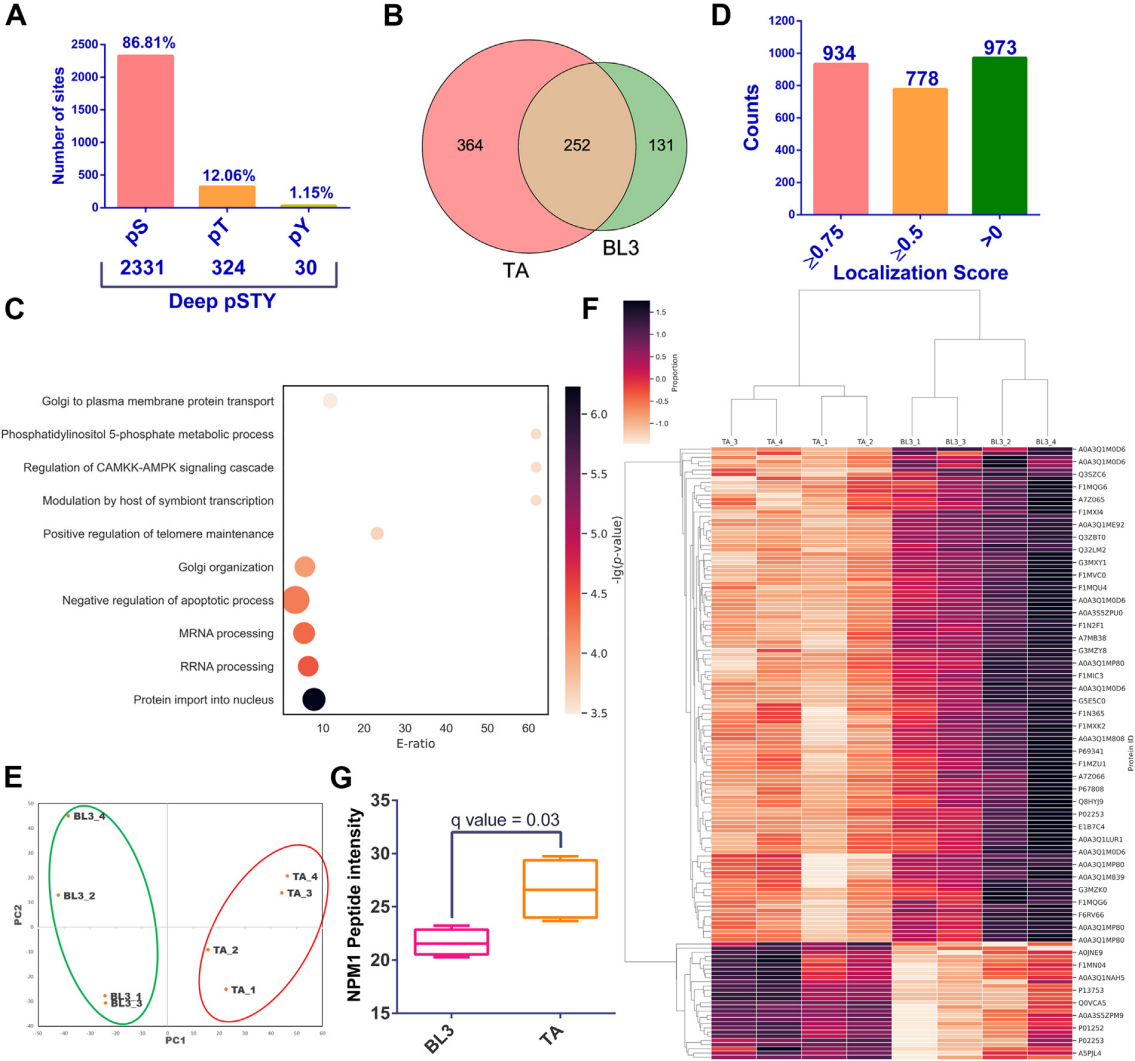
**Global phosphoproteome analysis of *T. annulata*–infected cells.***A*, the bar graph illustrates the distribution of modified sites across identified phosphosites. *B*, Venn diagram depicting the distribution of detected phosphoproteins in BL3 and TA cells. The chart illustrates the total number of identified proteins in each group, as well as the number of proteins shared between them. *C*, bubble chart displaying the top 10 enriched biological pathways in the TA cell-specific phosphoproteome, as determined by gene ontology enrichment analysis. The X-axis represents the E-ratio (enrichment ratio), while the Y-axis represents the *p*-value. Each bubble size corresponds to the significance of the pathway enrichment. *D*, bar graph depicting the localization scores for identified phosphosites. Phosphosites with a localization score of ≥0.75 are classified as class I phosphosites. *E*, principal component analysis (PCA) plot illustrating clustering patterns of samples. Each sample is represented as a separate point, highlighting the differential phosphorylation profiles between the two groups. *F*, heatmap illustrating ANOVA significant differentially regulated phosphoproteins in uninfected and infected samples. The heatmap also showcases the hierarchical clustering of samples based on their phosphorylation profiles. *G*, box plot depicting the normalized peptide intensity of NPM1(S70) in BL3 and TA cells, with corresponding q-values derived from the multiple ANOVA test.

Our investigation employed DIA coupled with label-free quantification to comprehensively analyze *T. annulata*–induced cellular signaling in lymphocytes. This approach enabled identifying and quantifying proteins from infected (TA) and noninfected (BL3) cells (supplemental Fig. S1*A*). The high quality and reproducibility of the data were confirmed by a strong correlation coefficient (R = 0.83) between biological replicates (supplemental Fig. S1*B*). Analysis of the host phosphoproteome indicated a substantial enrichment of phosphoserine (pS) residues (86.81%), with phosphothreonine (pT, 12.06%) and phosphotyrosine (pY, 1.15%) present at lower levels (Fig. 1*A*). The web logo (supplemental Fig. S1*C*) further visualizes the frequency of these modifications at specific amino acid positions (S/T/Y). Interestingly, most proteins (65%) harbored modifications at a single site, with a decreasing proportion exhibiting modifications at multiple sites (supplemental Fig. S1*D*). The analysis identified a total of 616 and 383 phosphorylated proteins in TA and BL3 cells, respectively (Fig. 1*B*, supplemental Table S1). Notably, 364 proteins were unique to TA cells, suggesting parasite-induced phosphorylation events. GO analysis of these uniquely phosphorylated proteins highlighted potential pathways specifically regulated during infection, including negative regulation of apoptosis and positive regulation of telomere maintenance (Fig. 1*C*). These findings suggest *T. annulata* may manipulate host cell survival and telomere integrity. We leveraged PTM scores to elucidate differentially phosphorylated events. Stringent criteria were implemented to define a high-confidence set of class I phosphosites (n = 934) corresponding to 400 proteins based on a localization probability threshold of ≥0.75 (Fig. 1*D*). Principal component analysis confirmed distinct clustering patterns between BL3 and TA samples based on these phosphosites (Fig. 1*E*). An additional filter was incorporated, requiring the presence of the modified site in at least 50% of the samples within each group. This resulted in 433 class I phosphopeptides qualifying for further analysis. Subsequent analysis using ANOVA identified 147 significantly altered phosphopeptide (supplemental Table S2). A heat map (Fig. 1*F*) visualizes the expression patterns of these differentially phosphorylated peptides. Examples of upregulated protein in TA cells include E3 ubiquitin-protein ligase (TRIP12), nucleophosmin (NPM1), lymphocyte cytosolic protein 1 (LCP1), Pinin (PNN), and dual-specificity kinase (DYRK1B). For example, the phosphorylation intensity of NPM1 at serine 70 is depicted in Figure 1*G*. These findings provide a comprehensive overview of the dynamic phosphorylation landscape induced by *T. annulata* infection. Identifying differentially phosphorylated proteins paves the way for further investigation into their functional roles during infection.

### Dissecting *T. annulata*'s Impact on Host Cell Response through Phosphoproteomics

**Supplemental Figure 2:**
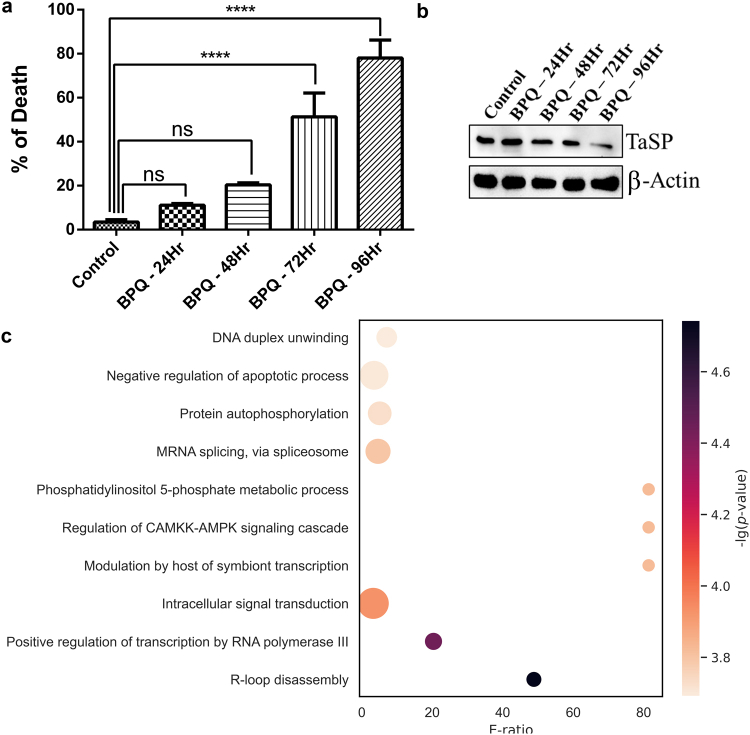


To dissect the intricate interplay between *T. annulata* signaling pathways and host cell posttranslational regulation, a targeted pharmacological approach was employed to achieve parasite clearance in infected lymphocytes. This strategy facilitated a comprehensive analysis of the parasite's impact on the host phosphoproteome and the subsequent host cellular response following parasite elimination. Infected lymphocytes (TA cells) were treated with BPQ (200 ng/ml), a well-established anti-theilerial drug (24). To assess cell viability over time, a trypan blue exclusion assay was performed. This assay revealed a progressive increase in cellular death, with statistically significant effects observed at 72 and 96 h posttreatment (supplemental Fig. S2*A*). Concomitant Western blotting analysis corroborated successful parasite clearance, evidenced by a reduction in TaSP (TA17315) expression at these time points (supplemental Fig. S2*B*). Based on these findings, the 96h time point was chosen for subsequent comparative phosphoproteomic analysis of treated (TA-BPQ) and untreated (TA) cells.

**Fig. 2:**
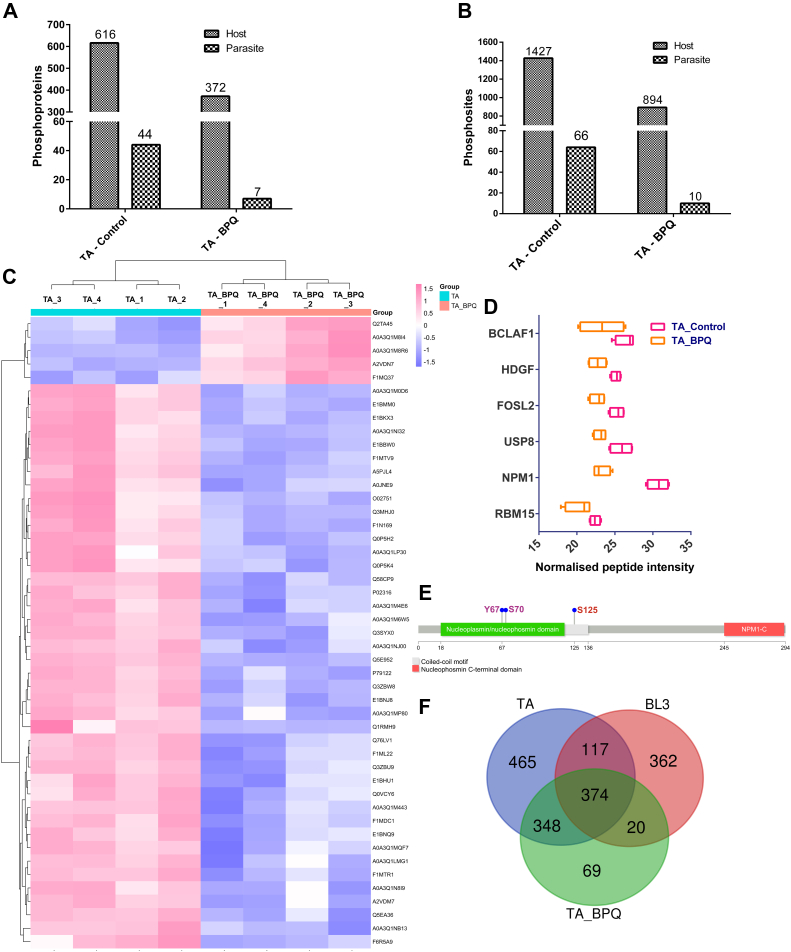
**Impact of parasite clearance on the phosphoproteome of *T. annulata*–transformed lymphocytes.***A*, bar graph depicting the total number of identified phosphoproteins in both host and parasite samples. *B*, bar graph illustrating the total number of detected phosphosites in both host and parasite samples. *C*, heatmap with hierarchical clustering presenting the expression profile of host phosphoproteins. *D*, horizontal box plot representing the normalized peptide intensity of selected host proteins found to be significantly downregulated upon parasite clearance, as evident from the multiple ANOVA test. *E*, lollipop plot illustrating the distribution of phosphorylation sites identified for NPM1. The Y-67 and S-70 sites are located within the nucleophosmin domain, while S-125 is part of the coiled-coil motif. *F*, Venn diagram showing the comparison of total phosphorylation sites among BL3, TA, and TA_BPQ samples.

We leveraged the established DIA-LFQ workflow for a comparative phosphoproteomic analysis. This approach aimed to identify changes in phosphorylation patterns and expression levels of individual phosphopeptides in both host and parasite proteomes. Four replicates per condition were used to ensure robust statistical analysis. A total of 616 host proteins with 1427 phosphorylation sites were identified in TA cells. Upon BPQ treatment, 244 proteins (533 phosphorylation sites) exhibited a loss of phosphorylation, while 34 gained new phosphorylation sites (Fig. 2, *A* and *B*, supplemental Table S3). Functional enrichment analysis on dephosphorylated host proteins revealed diverse cellular processes impacted by BPQ treatment, including DNA replication, apoptosis regulation, protein modification, RNA splicing, signal transduction, and symbiosis regulation (supplemental Fig. S2*C*). Interestingly, some of these processes, like regulation of CAMKK-AMPK signaling and negative regulation of apoptotic processes, were also enriched in the phosphoproteome unique to *T. annulata*–infected cells compared to uninfected controls (BL3). Furthermore, differential expression analysis identified 57 host cell phosphosites significantly regulated upon parasite clearance (Fig. 2*C*, supplemental Table S4). Key downregulated proteins included RNA-binding motif protein 15, NPM1, ubiquitin-specific peptidase 8 (USP8), FOSL2 (FRA2), hepatocyte growth factor (HDGF), and BCL2-associated factor 1 (BCLAF1). Notably, NPM1, previously observed to be upregulated in TA cells, displayed a decrease in phosphorylation (Y-67 and S-70) and protein expression upon parasite clearance, (Fig. 2, *D* and *E*). This suggests that these phosphorylation events might be crucial for NPM1 stabilization.

**Supplemental Figure 3:**
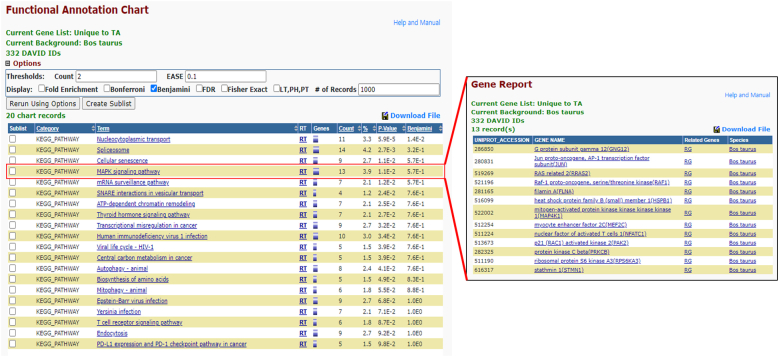
Functional annotation chart

We also analyzed all three datasets (BL3, TA, and TA-BPQ) and revealed a distinct population of 465 phosphosites unique to TA cells (Fig. 2*F*). These infection-specific phosphorylation events likely represent the parasite's crucial regulatory mechanisms to manipulate host cell processes and establish a favorable environment for survival and replication. To unravel the functional significance of these unique phosphorylation events, we performed gene enrichment analysis on the corresponding proteins (n = 334) utilizing the DAVID bioinformatics tool. This analysis yielded critical insights into the biological processes potentially modulated by *T. annulata* infection (refer to supplemental Fig. S3). The top five significantly enriched KEGG pathways included nucleocytoplasmic transport, spliceosome machinery, cellular senescence, MAPK signaling pathway, and mRNA surveillance pathways. The enrichment of these pathways suggests that *T. annulata* infection disrupts a multitude of cellular processes critical for host cell function. This orchestrated manipulation likely creates an environment conducive to parasite persistence within the host cell.

### Identification of Phosphorylation Sites from *T. annulata* Schizont Stage

**Fig. 3:**
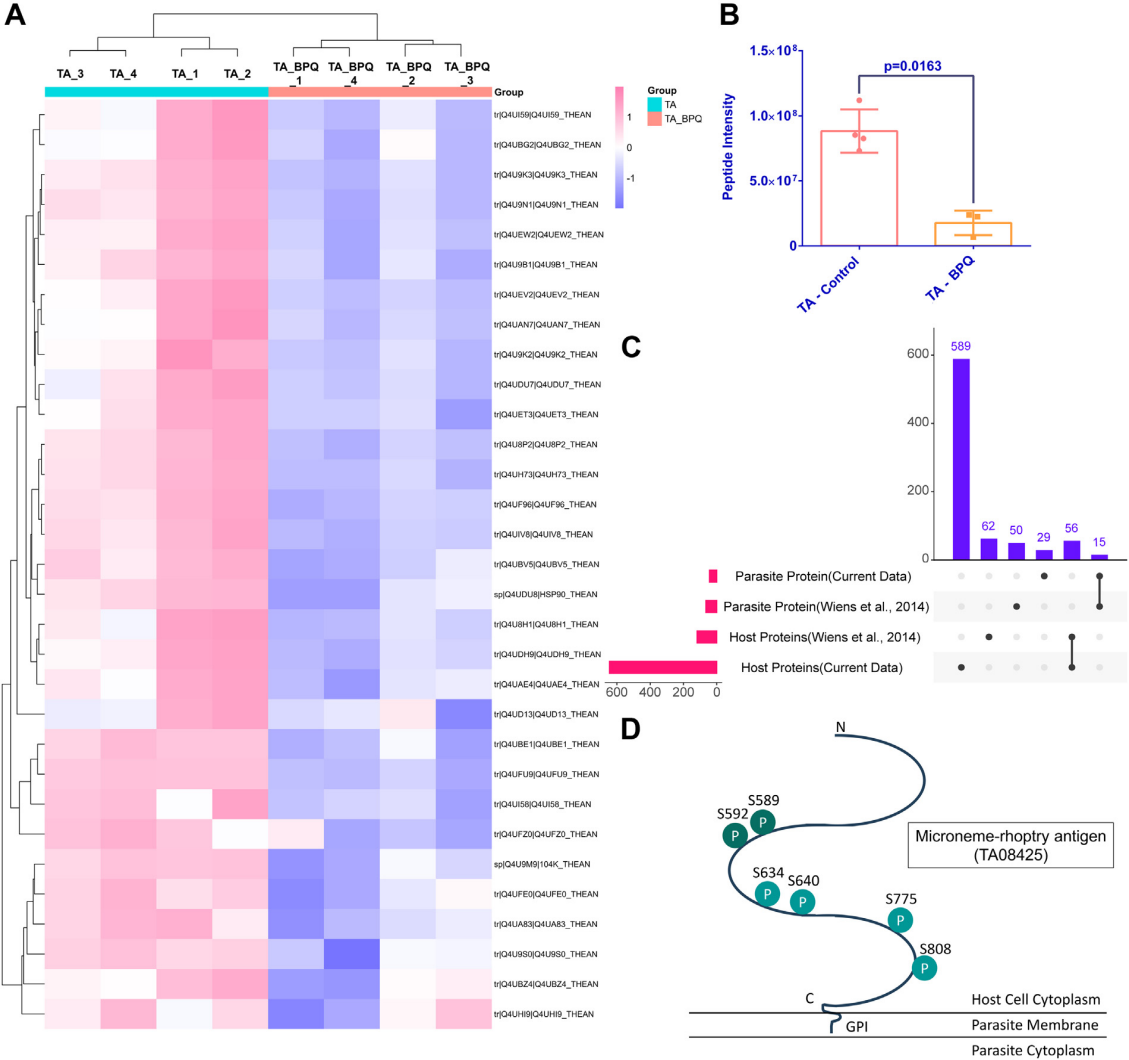
**Impact of parasite clearance on the phosphoproteome of *T. annulata*.***A*, heatmap with hierarchical clustering presenting the expression profile of parasite phosphoproteins. *B*, bar graph representing the LFQ peptide intensity of TA08425. A significant decrease in intensity is observed, denoted by a *p*-value of 0.0163. *C*, UpSet R plot compares our phosphoproteome data to (3). Overlapping proteins, categorized by host or parasite, are shown. Bar charts display protein counts, while *connecting lines* indicate shared proteins. *D*, detected phosphorylation sites on TA08425 are indicated by their respective amino acid numbers. Persian *green* circles (P) represent sites found in both studies (S634, S640, S775, S808), while Watercourse circles (P) highlight unique sites (S589, S592).

The parasite phosphoproteome analysis revealed distinct phosphorylation site distribution patterns. Phosphoserine residues constituted the predominant phosphorylation type (80%), followed by phosphothreonine (17.33%) and phosphotyrosine (2.66%). A comparative analysis revealed a significant downregulation of 48 phosphorylation sites across 32 parasite proteins following BPQ treatment (Fig. 3*A*, supplemental Tables S5 and S6). Notably, several key parasite proteins demonstrated a concurrent loss of phosphorylation and expression, including a microneme-rhoptry antigen (TA08425), cyclophilin (TA14055), splicing factor (TA06100), probable ATP-dependent 6-phosphofructokinase (TA13950), and RNA helicase (TA04765) (Fig. 3*A*). The decreased peptide intensity of TA08425 (104 kDa microneme/rhoptry antigen) in TA-BPQ compared to TA cells is depicted in Figure 3*B*. For broader context, we compared our parasite phosphoproteomic data with the study by (3), which focused specifically on parasite proteins (Fig. 3*C*). Notably, our analysis of whole-cell lysate revealed some unique proteins compared to their schizont-enriched dataset. While potential sequence variations precluded a direct site-by-site comparison, we identified phosphorylation on the parasite protein TA08425 at six distinct sites. Four of these sites (S634, S640, S775, and S808) corroborated findings by Weins *et al*. (2014). Importantly, our data revealed two novel phosphorylation sites for this protein: S589 and S592 (Fig. 3*D*).

### Phosphoproteomics-Guided Kinome Analysis: A Computational Approach Identifies Key Kinase Families in Host and Parasite

Infection with *T. annulata* significantly disrupts various host cell signaling pathways, as reflected by the enrichment of distinct signaling pathways within the identified phosphoproteome. Kinases are well-established master regulators of these pathways, and their dysregulation is implicated in numerous human diseases, including cancer. We employed in-silico kinase prediction utilizing the iGPS to gain deeper insights into the upstream regulatory mechanisms governing these phosphorylation events. This computational tool leverages two complementary approaches: analysis of known kinase recognition motifs and protein–protein interaction databases. This combined strategy allows iGPS to predict kinases with the highest likelihood of phosphorylating specific protein sites. Next, we analyzed the regulatory landscape of kinases during *T. annulata* infection by leveraging phosphoproteomic analysis of both host and parasite components.

**Fig. 4:**
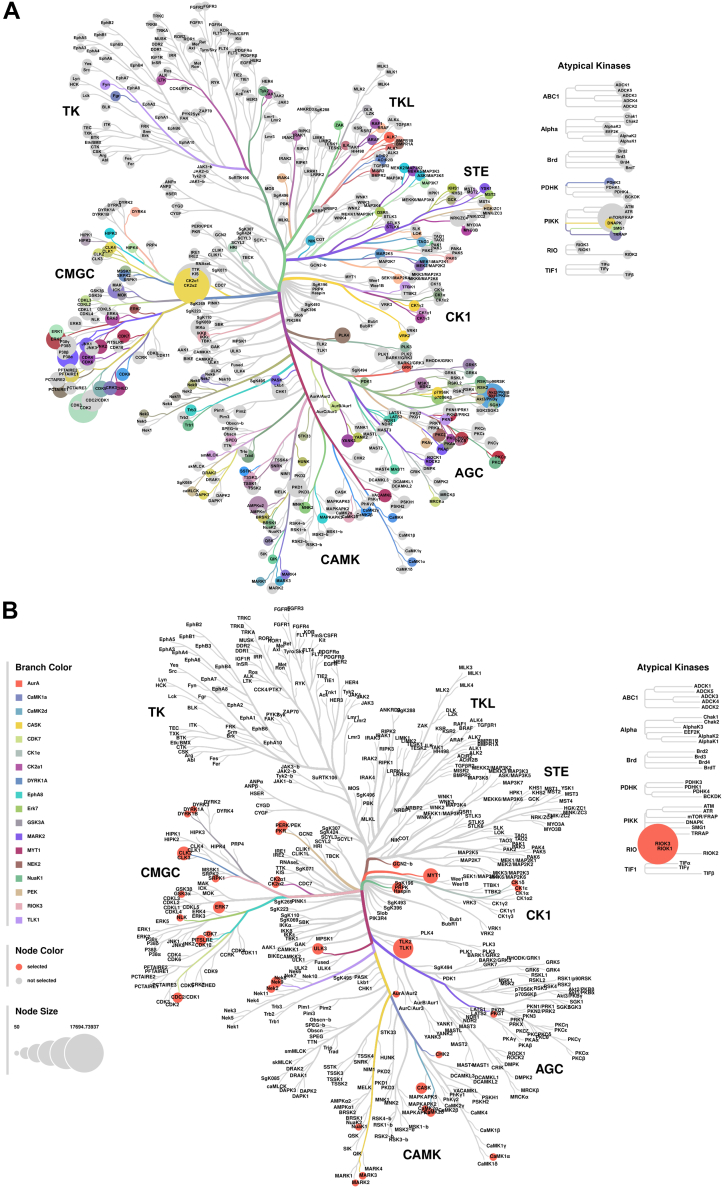
**Characterization of *T. annulata*–induced kinase regulation.***A*, kinase tree map displaying the predicted upstream kinases for host phosphosites using iGPS 1.0.1 that lost phosphorylation upon parasite clearance. Each kinase was assigned a weightage value as described in the Experimental procedures section and mapped onto the human kinome tree using CORAL. Node size corresponds to the weightage of the kinase. *B*, kinase tree map illustrating the predicted upstream kinases for parasite phosphosites using GPS 6.0. Node size indicates the weightage of the kinase.

Specifically focusing on the host phosphoproteome, we investigated proteins exhibiting dephosphorylation following BPQ treatment. Using iGPS 1.0.1 predicted the kinases likely responsible for the observed phosphorylation events across 533 sites on 244 distinct proteins (supplemental Table S7). Subsequently, we mapped these predicted kinase–substrate interactions onto the human kinome tree. This analysis revealed a diverse array of potentially involved kinase families, encompassing CMGC (e.g., CK2A, ERK1/ERK2, CDK2), CAMK (e.g., AMPKα1, AMPKα2, PKD1), AGC (e.g., AKT1, AKT2), CK1 (e.g., TTBK1, TTBK2, CK1α2), STE (e.g., MEK1, MEK2, YSK1), TKL (e.g., RAF1, BRAF1), TK (e.g., LTK, Fyn, Fgr), atypical kinases (e.g., FRAP, DNAPK), and others (complete list). Interestingly, the CMGC family emerged as the most overrepresented group among the predicted kinase regulators (Fig. 4*A*).

Given the considerably smaller kinome repertoire of *T. annulata* compared to the human host (52 reported kinases versus a much larger human kinome), a homology search was conducted to identify potential human counterparts for these parasite kinases. Notably, no tyrosine kinases were identified within the *T. annulata* kinome. The GPS 6.0 tool and its curated kinase–substrate relationship data were employed to predict upstream regulators responsible for parasite phosphoproteome modifications. A weightage score reflecting the potential importance of each predicted *T. annulata* kinase was assigned based on the number of predicted phosphorylation targets (lower score indicates higher potential significance). This data was then visualized using the CORAL kinome visualization tool (Fig. 4*B*, supplemental Table S8). The analysis revealed a diverse array of potential kinase families influencing parasite signaling pathways, including CMGC (e.g., CLK2, ERK7, CDC2), CAMK (e.g., CaMK2α, CASK), and atypical kinases (e.g., RIOK3). Like the host analysis, the CMGC family emerged as the most prominent group among the predicted parasite kinase categories (Fig. 4*B*). This intriguing observation suggests a potentially conserved role for CMGC kinases in regulating host and parasite signaling during *T. annulata* infection.

In conclusion, this phosphoproteomic exploration underscores the critical role of kinase families, particularly those belonging to the CMGC group, in regulating the intricate interplay between host and parasite signaling pathways during *T. annulata* infection.

### *T. annulata* Infection Modulates Host Transcriptional Response by Phosphorylation of Key TFs

**Fig. 5:**
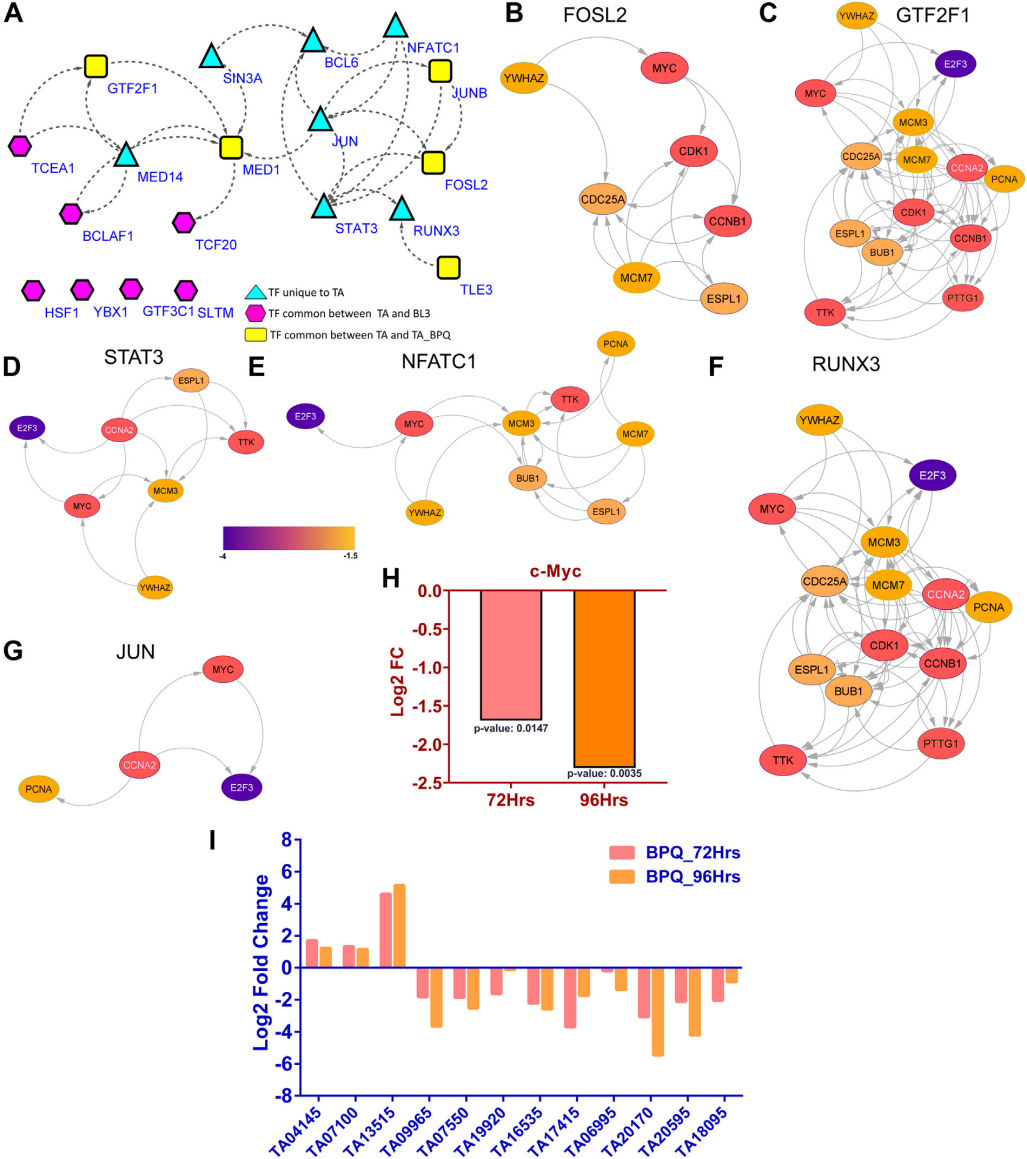
**Integrating *T. annulata* responsive phosphoproteome data with transcriptome for transcription factor analysis.***A*, STRING network visualizing interactions among transcription factors (TFs) identified in the phosphoproteome dataset. The different shapes and colors represent distinct categories of TFs based on their phosphorylation status and presence in TA or BL3 cells. TFs that are unique to TA cells and lose phosphorylation upon BPQ treatment are depicted as triangles filled with *cyan*. TFs that are shared between TA and BL3 cells are represented as hexagons filled with *magenta*. TFs that are exclusively found in TA cells and do not lose phosphorylation upon BPQ treatment are shown as squares filled with *yellow*. These categorizations highlight the differential regulation of TFs in response to BPQ treatment. *B*–*G*, the STRING network visualizations depict the downstream genes regulated by each transcription factor (TF) (*B*: FOSL2, *C*: GTF2F1, *D*: STAT3, *E*: NFATC1, *F*: RUNX3, *G*: JUN). The downstream genes were curated from publicly available human regulatory databases, including ENCODE, CHEA, and MotifMap4, ensuring the inclusion of experimentally validated or computationally predicted TF-target interactions. In each network, nodes represent the downstream genes regulated by the respective TF, and edges indicate interactions among these genes. The networks do not depict direct TF-target binding but rather the relationships between the downstream genes influenced by each TF. The fill color of each node corresponds to the log2 fold-change expression of the gene, derived from RNA-seq data comparing TA versus TA_BPQ at 96 h post-BPQ treatment. The analysis specifically focuses on downregulated genes, with the color intensity reflecting the degree of downregulation. The color gradient ranges from −1.5 to −4 log2 fold change, with the corresponding scale displayed between panels (*D*) STAT3 and (*E*) NFATC1 for reference. The networks were generated using Cytoscape. *H*, bar graph illustrating the log2 fold change values of c-MYC expression from RNA-seq analysis at 72 and 96 h post-BPQ treatment, compared to untreated TA cells. The expression of c-MYC decreases over time, with a log2 fold change reduction at both time points. *p*-values were calculated using DESeq, with statistical significance observed at 72 h (*p* = 0.0147) and 96 h (*p* = 0.0035). *I*, bar graph illustrating the expression changes of *T. annulata* transcription factors identified from RNA-Seq data at 72 and 96 h post-BPQ treatment, compared to untreated TA cells. The analysis identified 12 putative parasite TFs, including eight from the ApiAP2 family. Five ApiAP2 TFs exhibited downregulation, while three were upregulated upon BPQ treatment. The four remaining non-ApiAP2 TFs were consistently downregulated. Expression levels are shown as log2 fold change values, with statistical significance assessed using DESeq.

**Supplemental Figure 4:**
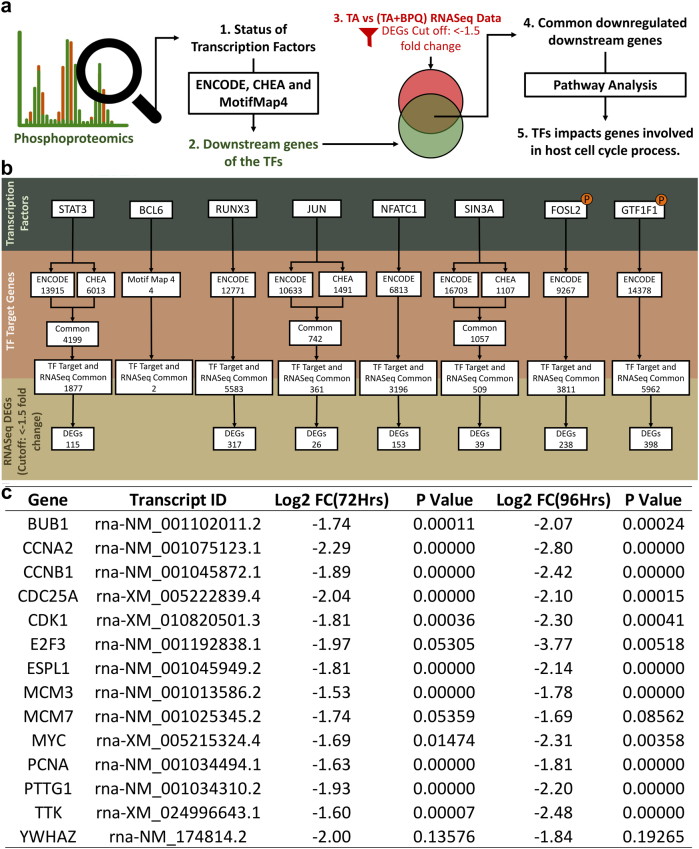


Building on our phosphoproteomic analysis that revealed a multitude of phosphorylated proteins, we delved deeper into their functional implications. Given the critical role of TFs in gene regulation and the established effects of phosphorylation on TF activity, we specifically investigated phosphorylated TFs within *T. annulata*–infected cells. We identified a total of 19 TFs within *T. annulata*–infected cells (TA) compared to the uninfected control group (BL3). Notably, 12 of these TFs exhibited distinct phosphorylation patterns upon infection, including MED14, SIN3A, BCL6, cJUN, NFATC1, STAT3, RUNX3, GTF2F1, MED1, JUNB, TLE3, and FOSL2 (Fig. 5*A*). Interestingly, subsequent treatment with BPQ resulted in the dephosphorylation and concomitant downregulation of seven of these TFs (MED14, SIN3A, BCL6, cJUN, NFATC1, STAT3, and RUNX3, but not JUNB). This observed correlation between phosphorylation status and protein abundance suggests a potential regulatory role for phosphorylation in governing TF functionality during *T. annulata* infection. To delve deeper into the downstream consequences of dysregulated TF activity, RNA-Seq was performed on both BPQ-treated (72 h & 96 h) and control cells (supplemental Table S9). Given the limited availability of established target genes for bovine TFs, human databases such as ENCODE, CHEA, and MotifMap4 were employed to identify potential downstream targets for eight of the identified TFs (STAT3, BCL6, RUNX3, cJUN, NFATC1, SIN3A, FOSL2, and GTF2F1) (supplemental Fig. S4, *A* and *B*). Subsequent gene enrichment analysis utilizing DAVID revealed that cell cycle regulation emerged as a prominent pathway commonly impacted by all TFs except SIN3A (supplemental Fig. S4*C*). Differential expression analysis identified a significant downregulation in the transcripts of genes known to be regulated by these TFs (STAT3, BCL6, RUNX3, cJUN, NFATC1, FOSL2, and GTF2F1) after parasite clearance (Fig. 5, *B*–*G*). Further investigation identified cMYC as a shared downstream target gene regulated by all seven TFs, with its expression gradually decreasing upon parasite clearance. RNA-seq analysis confirmed these results, showing a gradual time-dependent decrease in cMYC expression after BPQ administration. (Fig. 5*H*).

Although the phosphoproteomic analysis did not identify parasite-specific TFs, the RNA-Seq data provided valuable information (supplemental Table S10). By interrogating the parasite transcriptome for genes annotated with the GO term “sequence-specific DNA binding transcription factor activity” (GO:0003700), 12 putative parasite TFs were identified. Notably, eight of these TFs belonged to the ApiAP2 family, characterized by the presence of the AP2 domain (Fig. 5*I*). Interestingly, treatment with the anti-theilerial drug BPQ resulted in differential expression patterns among these parasite TFs. Five members of the ApiAP2 family were downregulated, while three exhibited upregulation. The remaining four TFs, not belonging to the ApiAP2 family, all displayed downregulation upon BPQ treatment (Fig. 5*I*).

This integrated phosphoproteomic and RNA-Seq approach sheds light on a novel regulatory mechanism employed by *T. annulata*. The parasite manipulates host cell signaling through targeted phosphorylation of TFs, potentially influencing the cell cycle pathway and cMYC expression. Additionally, the identification of parasite TFs opens avenues for future investigations into their role in parasite biology and potential therapeutic targets (25).

### ERK1/2 Signaling Pathway Mediates Host Transcriptional Response During *T. annulata* Infection

**Fig. 6:**
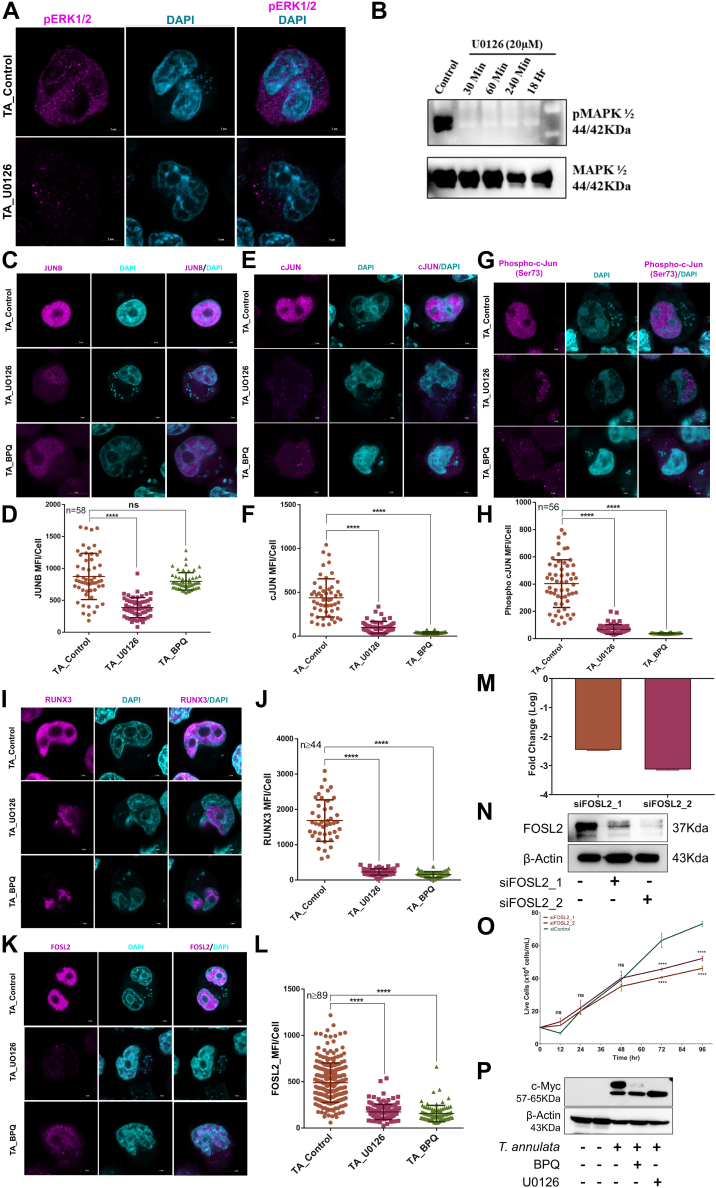
**ERK regulates transcription factors FOSL2, cJUN, JUNB, and RUNX3.***A*, immunofluorescence assay (IFA) showing the expression of pERK1/2 (*magenta*) in TA cells and TA cells treated with the MEK inhibitor U0126 for 48 h at 10 μM, alongside nuclear staining with DAPI (*turquoise*). *B*, immunoblot showing the expression of both pERK1/2 and wtERK1/2 in TA cells treated with U0126 at various time points up to 48 h. *C*–*L*, immunofluorescence assay (IFA) showing the expression of JUNB, cJUN, cJUN (S73), RUNX3, and FOSL2 in TA cells, TA cells treated with U0126 (10 μM for 48 h), and TA cells treated with BPQ (200 ng/ml for 48 h). The intensity quantification of the aforementioned TFs from the IFA is represented for all three conditions, with each dot representing a single cell in the graph. Statistical significance was assessed using one-way ANOVA, with ∗∗∗∗ indicating a *p*-value <0.0001. *M*, qRT-PCR analysis showing the log2 fold change expression of FOSL2 mRNA levels upon siRNA inhibition of FOSL2 in TA cells using two different siRNA constructs at 96 h post-transfection. The results are visualized using a bar graph, with the relative fold change value quantified using a scrambled RNA construct as the control. *N*, immunoblot showing the impact of siRNA inhibition on the protein level of FOSL2. β-Actin was used as a loading control. *O*, line graph showing TA cell proliferation upon siRNA inhibition of FOSL2. Cell counts were determined using the trypan *blue* dye exclusion method at 24-h intervals post-transfection. The y-axis represents the live cell population. Statistical significance was assessed using one-way ANOVA, with ∗∗∗∗ indicating a *p*-value <0.0001. *P*, immunoblot showing the expression of c-MYC in various conditions, with ß-actin used as a loading control. The expression was assessed in uninfected healthy PBMC (hPBMC), *T. annulata*–infected cells (TA), and TA cells treated with U0126 (10 μM for 48 h) and BPQ (200 ng/ml for 48 h).

*In silico* analysis predicted ERK1/2 kinases as potential regulators of five TFs: FOSL2, cJUN, JUNB, RUNX3, and BCL6 (supplemental Fig. S5*A*). This finding contrasted with prior studies suggesting no role for ERK1/2 in *Theileria*-infected cells (14, 26, 27, 28). To reconcile this discrepancy, we employed commercially available antibodies specific for the activated form of ERK1/2(phosphorylated threonine 202/204). Both immunoblotting and immunofluorescence assaysIFA confirmed the presence and activation of ERK1/2 kinases within *T. annulata*–infected cells (Fig. 6, *A* and *B*). Furthermore, treatment with U0126, a well-established inhibitor of MEK, the upstream activator of ERK1/2, resulted in dephosphorylation of ERK1/2, validating the predicted role of these kinases in infected cells. We subsequently investigated the regulatory influence of ERK1/2 on the identified TFs. Antibodies targeting each TF (FOSL2, cJUN, phospho-cJUN (S73), JUNB, RUNX3, STAT3, and phospho-STAT3 (Y705)) were employed to assess protein expression and phosphorylation status. To strengthen the association between infection and TF regulation, we introduced additional experimental conditions: U0126 treatment to inhibit ERK1/2 and BPQ treatment to eliminate parasites.

Immunofluorescence analysis revealed prominent nuclear localization of FOSL2, RUNX3, JUNB, cJUN, and phospho-cJUN (S73) in *T. annulata*–infected cells (Fig. 6, *C* and *L*). Notably, inhibition of ERK1/2 with U0126 resulted in a significant decrease in the expression of these TFs. Similarly, parasite clearance via BPQ treatment mirrored the effect of U0126, demonstrating a reduction in all aforementioned TFs. Both STAT3 and phospho-STAT3 (Y705) were detected in infected cells; however, consistent with our in-silico predictions and prior observations, ERK1/2 inhibition did not affect STAT3 expression or phosphorylation (supplemental Fig. S5*B*). These findings bridge the gap in the existing literature and establish ERK1/2 as critical regulators of specific TFs during *T. annulata* infection. The activation of ERK1/2 appears to influence either directly or indirectly the expression and/or phosphorylation of FOSL2, cJUN, JUNB, and RUNX3, potentially impacting their transcriptional activity and the subsequent host cell response. To elucidate the functional role of FOSL-2, a key AP1 transcription factor, we employed siRNA-mediated knockdown (Fig. 6, *M* and *N*). This approach revealed FOSL-2's critical role in both host cell proliferation and survival (Fig. 6*O*). These observations collectively suggest that FOSL-2 plays a vital part in the parasite's life cycle. Furthermore, inhibiting FOSL-2 likely disrupts a crucial pathway for parasite proliferation, potentially leading to a diminished parasite burden. To validate the *in silico* and transcriptome data suggesting a link between c-Myc and ERK1/2 signaling, we treated cells with U0126, an ERK1/2 inhibitor, and monitored c-Myc expression. U0126 treatment resulted in reduced c-Myc levels, supporting the hypothesis that ERK1/2 signaling plays a role in activating c-Myc (Fig. 5*P*). These findings demonstrate that host ERK1/2 signaling is necessary for c-Myc activation.

Our study highlights the crucial role of ERK1/2 signaling in survival of the *Theileria*-infected cells. This pathway plays a central role in regulating parasite physiology by orchestrating directly or indirectly the phosphorylation of key transcription factors, including RUNX3, FOSL2, BCL6, c-JUN, JUNB, and c-Myc.

### ERK1/2 Inhibition Impairs *T. annulata* Proliferation and Triggers Host Cell Apoptosis

**Fig. 7:**
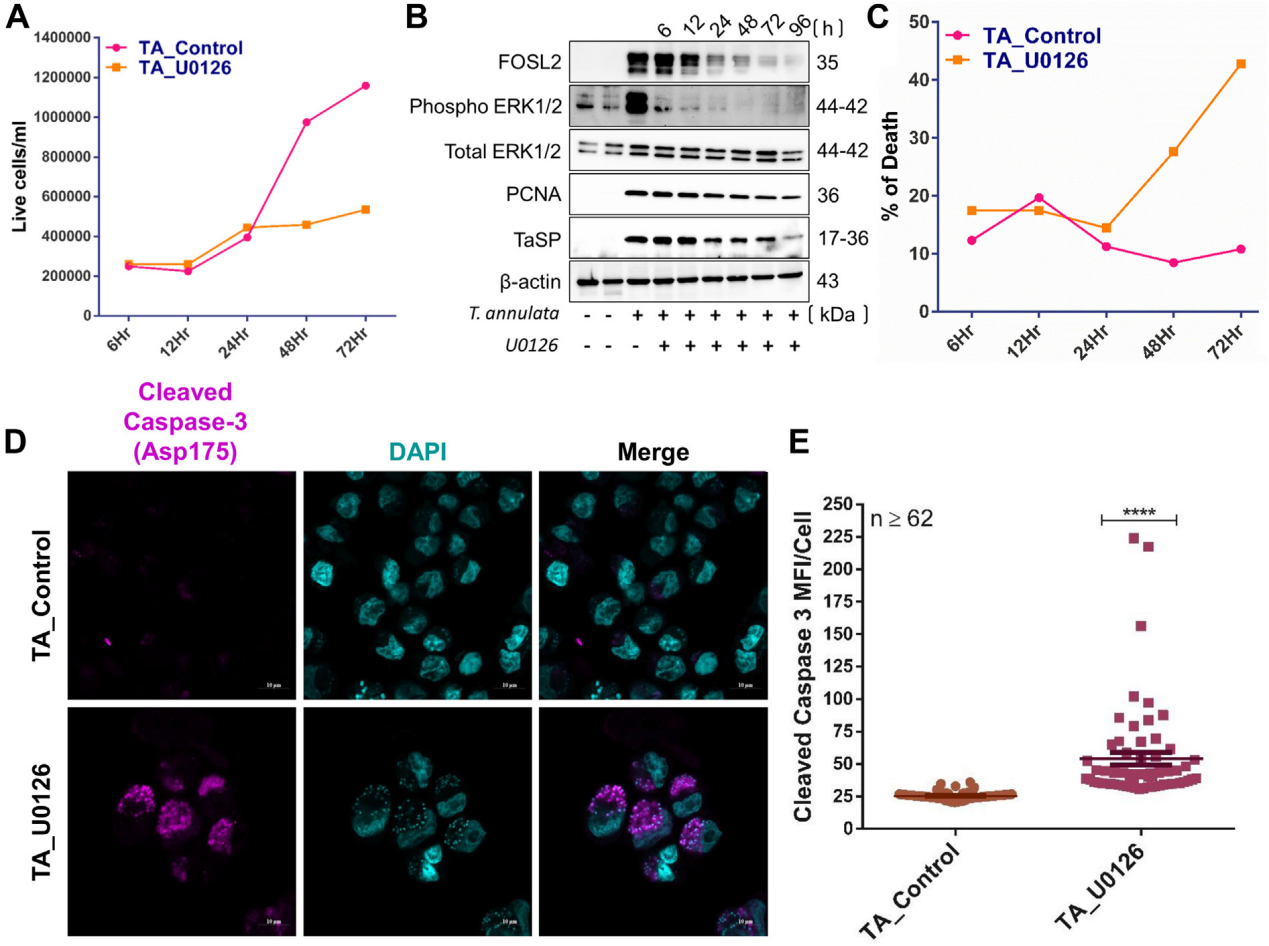
**ERK inhibition leads to decrees TA cell proliferation followed by apoptosis.***A*, graph showing the total live cell count in TA control and U0126-treated cells, measured using the trypan blue dye exclusion method. Each time point represents the mean population from three observations. *B*, immunoblot displaying the expression of FOSL2, pERK1/2, ERK1/2, PCNA, TaSP, and β-actin in untreated TA cells and TA cells treated with U0126 at various time points. *C*, graph depicting the percentage of cell death in TA cells treated with U0126, along with nontreated controls, across different time points. *D*, immunofluorescence assay (IFA) showing cleaved caspase-3 (Asp175) (*magenta*) in TA cells and TA cells treated with U0126 (10 μM for 48 h). Nuclei are stained with DAPI (*turquoise*). Images were captured in Airyscan mode and processed using Zen *Black*, with quantification performed in Zen *Blue* edition. *E*, graph presenting cleaved caspase-3 (Asp175) intensity per cell, derived from IFA images, for both TA cells and U0126-treated TA cells. Statistical analysis was performed using an unpaired *t* test. ∗∗∗∗ indicates a *p*-value <0.0001.

To delineate the functional consequences of ERK1/2 inhibition, we assessed its impact on both *T*. *annulata* (TA) cell proliferation and host cell viability. A trypan blue exclusion assay revealed that U0126 treatment effectively halted TA cell proliferation by 24 h compared to untreated controls with sustained growth (Fig. 7*A*). This confirms U0126's ability to suppress TA cell expansion. Western blot analysis of PCNA, a marker of ongoing cellular proliferation, further corroborated this effect. U0126 treatment caused a time-dependent decrease in PCNA expression, solidifying its role in suppressing TA cell proliferation (Fig. 7*B*). Immunoblotting the parasite-specific protein TaSP showed a time-dependent reduction following U0126 treatment (Fig. 7*B*), suggesting a decline in parasite burden within infected cells. Additionally, cell death assays revealed a significant increase in dead TA cells within 48 h of U0126 treatment, reaching nearly 40% by 72 h (Fig. 7*C*). This aligns with the observed upregulation of cleaved caspase-3 (Asp175), a marker of apoptosis. Immunofluorescence analysis confirmed U0126-induced apoptosis, with cleaved caspase-3 (Asp175) levels substantially increased in treated cells at 48 h post-treatment compared to undetectable levels in untreated cells (Fig. 7, *D* and *E*). Collectively, these findings demonstrate that U0126 disrupts ERK1/2 signaling, leading to a multipronged effect: (1) inhibition of TA cell proliferation, (2) reduction in parasite burden, and (3) induction of host cell apoptosis. This highlights the critical role of ERK1/2 in promoting survival and proliferation pathways within TA cells.

## Discussion

By employing global phosphoproteomics, this study aimed to shed light on the complex interplay between parasite and host cell signaling pathways. We performed an integrated global phosphoproteomic and transcriptomic analysis, revealing the striking impact of *T. annulata* on host cell signaling and cellular behavior. Identified phosphorylated proteins were used to generate a *T. annulata*–responsive kinome network. Based on this, we identified the phosphorylation status of regulated transcription factors that are either direct or indirect MAPK/ERK substrates and demonstrated their important role in the resulting phenotype. The results reveal a substantially greater range of *T. annulata*–regulated signaling cascades than previously appreciated and they provide a resource for generating deeper insights into their role in pathogenesis and potential host cell transformation. Deciphering the parasite's manipulation of the host kinome promises to advance our understanding of *T. annulata* pathogenesis and pave the way for the development of novel therapeutic strategies for combating tropical theileriosis.

While prior research has investigated global phosphorylation events in *T. annulata* schizont proteins, a critical knowledge gap persists in our understanding of host proteins and signaling used by the parasite during infection (3). We performed a phosphoproteomic analysis of infected, noninfected, and cured leukocytes to elucidate *Theileria*-induced signaling pathways. We performed a DIA for the analysis, allowing higher sensitivity and protein coverage than the classic data-dependent acquisition (29). Building on prior evidence that *T. annulata* parasites disrupt host leukocyte signaling via altered kinase activity and protein phosphorylation (6), this study identified 465 host class 1 phosphorylation sites significantly modulated by parasite infection. Pathway analysis revealed the MAPK signaling pathway as a critical player. The MAPK pathway is well-established for its influence on diverse cellular processes, including proliferation (30). Notably, 14 proteins within this network exhibited phosphorylation specifically in *Theileria*-infected cells, with these modifications disappearing upon parasite clearance. These findings strongly suggest that *T. annulata* actively manipulates the MAPK pathway, reinforcing previous research that showed the importance of MAPK in *Theileria* pathogenesis (3, 14, 28). Bioinformatic analysis of phosphorylation sites significantly modulated by parasite infection revealed enrichment in processes critical for cancer development. These include (i) negative regulation of apoptosis, (ii) CAMK-mediated regulation, and (iii) positive regulation of telomere maintenance. This highlights the parasite's potential to manipulate these signaling pathways. Notably, NPM1, an established oncogene, displayed parasite-dependent upregulation in infected cells (TA) compared to controls (BL3). Furthermore, our data revealed parasite-dependent phosphorylation of NPM1 at S70, a modification known to impact its stability in humans. CDKs are known to influence S70 phosphorylation and might play a role in *Theileria* infection (31). Therefore, future studies investigating targeted CDK inhibition in TA cells are warranted to elucidate their potential involvement in *T. annulata*–mediated regulation of NPM1.

*Theileria* infection is known to induce constitutive activation of specific host kinases, including PI3-K, JNK, CK2, and Src-family kinases (3, 6, 10, 11, 12, 13, 14, 15). These activated kinases are believed to be critical in regulating the transformed phenotype observed in infected cells. *In silico* analysis of predicted kinase–substrate interactions identified kinase families, including CMGC, TK, TKL, STE, CK1, AGC, and CAMK, as potential regulators of host proteins exhibiting unique phosphorylation patterns during *T. annulata* infection. Future studies will be crucial to elucidate the functional consequences of these phosphorylation events. Most of the regulated phosphoproteins and the predicted upstream kinases identified in the present study have not been previously associated with the response to *T. annulata* infection. However, we did find certain kinases which were previously reported, such as CKII and CDK, to be present in our data, reinforcing their role during *T. annulata* infection (3, 6, 16, 25, 32). Adding to previous research, we show CDK phosphorylation in *Theileria*-infected cells, particularly in CDK1NA, CDK12, CDK2, CDK11B, and CDK1. Building on the established role of PKM2 in *Theileria*-induced metabolic reprogramming (33), we identified phosphorylation at Ser-37 of PKM2 as a potential regulator. This modification might modulate the interaction between PKM2 and its stabilizer, TaPin1, thereby impacting HIF1α signaling (17). These findings suggest a novel mechanism in *Theileria* pathogenesis, warranting further investigation of this regulatory pathway. Our investigation of *T. annulata*–induced phosphoproteins revealed a panel of transcription factors, including FOSL2, GTF2F1, c-JUN, NFATC1, STAT3, and RUNX3. While STAT3's role in *Theileria* infections is well-established, the functions of the others (GTF2F1 and NFATC1) remain unclear (25). Our analysis identified phosphorylation of c-JUN(S63), FOSL2(S200), and JUNB(S251,255,259) during *T. annulata* infection. Cdk3-mediated phosphorylation of c-Jun at Ser63 is known to enhance cell transformation (34). *Theileria* infection induces a transformed phenotype in cells, which is attributed to the upregulation of Jun and Fos family members and the continuous activation of JNK signaling (28). While c-JUN(S63) phosphorylation has been previously reported for *Theileria*-infected leukocytes (28), phosphorylation of FOSL2 (S200) and JUNB (S251,255,259) appears to be a novel finding. RNA-seq identified a downregulation of cell cycle–regulated genes known to be targeted by these TFs (FOSL2, GTF2F1, c-JUN, NFATC1, STAT3, and RUNX3) on parasite clearance. Our transcriptome analysis offers the first comprehensive view of downstream genes associated with each transcription factor during *Theileria* infection. Notably, E2F3, a well-established cell cycle regulator, was downregulated following parasite clearance. Previous research by Tretina *et al*. (2020) has demonstrated that *Theileria* parasites manipulate E2F signaling to promote leukocyte proliferation (1). These combined findings highlight E2F signaling as a promising avenue for therapeutic intervention. Intriguingly, c-MYC emerged as a potential link between these six TFs (FOSL2, GTF2F1, c-JUN, NFATC1, STAT3, and RUNX3), despite not being identified in our phosphorylation analysis. This is relevant because c-MYC activation by *Theileria* promotes infected B-cell survival (25). *Theileria* infection normally triggers cytokine secretion such a GM-CSF^19^, activating TFs (STAT3, PI3-K, AP-1, and E2F) that drive c-MYC production. However, parasite death disrupts this pathway, leading to a combined decline in TF activity, c-MYC levels, and ultimately cell death (25). Our data suggests a more complex regulatory network for c-Myc activation during *T. annulata* infection, potentially involving multiple pathways beyond the established GM-CSF/JAK2/STAT3 signaling cascade (25).

Our bioinformatics identified ERK1/2, as the main MAPK kinase activated by *T. annulata* (35). Our findings suggest an ERK-dependent regulatory pathway for FOSL2, JUNB, and c-JUN in *Theileria*-infected cells. Interestingly, ERK1/2 deregulation is common in many cancers, promoting uncontrolled cell growth and survival. In contrast to our findings, previous research suggests MAPK family members, like JNK, and ATF2 are activated in *Theileria*-transformed cells, while ERK2 and p38 remain inactive (14, 26, 27, 28, 36). The discrepancy might also be attributed to the increased sensitivity of the ERK1/2 antibody used in our study. Our findings demonstrate that *Theileria*-mediated ERK phosphorylation triggers the subsequent direct or indirect phosphorylation of several transcription factors, including FOSL2, GTF2F1, c-JUN, JUNB, NFATC1, and RUNX3. Furthermore, inhibition of ERK signaling leads to the dephosphorylation of these transcription factors (FOSL2, c-MYC, c-JUN, JUNB, and RUNX3) and the subsequent induction of apoptosis in *T. annulata*–infected cells. This observation underscores the critical role of AP-1 signaling in *Theileria* pathogenesis, potentially mimicking features of cancer development. These findings suggest that targeting the ERK–AP-1 signaling axis holds promise as a therapeutic strategy against *T. annulata* infection, warranting further investigation.

This study also sheds light on the multifaceted strategies employed by *T. annulata* parasites to manipulate host cells and potentially evade immune defenses. We identified key parasite phosphorylated proteins and their potential cognate kinases, suggesting mechanisms by which *T. annulata* might hijack host cellular processes. Our analysis expands on previous research by identifying novel phosphorylation sites on the parasite protein p104 (TA08425), including one that regulates JNK2 activity (3, 13). These findings highlight the importance of p104 phosphorylation in parasite biology. Our study identified phosphorylation at Ser183 of TA14055, a putative *Theileria* cyclophilin (CYP) with peptidyl-prolyl cis-trans isomerase (PPIase) activity, a critical process for protein folding and trafficking (37, 38). Cyclophilins from parasites are attractive targets for drug development, but their specific function in *Theileria* remains unclear. Notably, a recent study found *T. annulata* cyclophilin1 (TA19600) interacts with host cell MED21, suggesting a potential role in mediating interactions between the parasite and host leukocytes (39). The presence of both TA14055 and TaPin1, another PPIase, within *T. annulata* suggests a potentially complex PPIase network (17). These findings highlight the need for further investigation into the functional role of TA14055, the impact of Ser183 phosphorylation, and the potential interplay between CyPs and TaPin1 in *Theileria*. While parasite TF phosphorylation was not detected, transcriptome analysis revealed upregulation of several TF families, including ApiAP2 and HAP complex factors. Our transcriptomic analysis unveiled significant modulations in the expression profiles of ApiAP2 transcription factors, recognized for their critical role in regulating developmental processes within Apicomplexan parasites (40). Notably, TA07550, an ApiAP2 factor exhibiting peak expression during the schizont stage, demonstrated a marked downregulation upon parasite elimination. This observation aligns with prior studies, highlighting the potential significance of TA07550 in parasite lifecycle (41). Further investigations into its downstream target genes and potential for targeted therapeutic intervention are highly warranted. Furthermore, our study identified a compelling finding: *T. annulata* exhibited elevated expression of Hap complex transcription factors during the schizont stage, followed by a significant downregulation upon treatment that disrupts ROS production. The Hap complex, well-established for its role in managing cellular stress responses in yeast (42), suggests a potential Achilles' heel for *Theileria*. This discovery presents an exciting opportunity for further investigation into the specific role of the Hap complex in *Theileria*'s stress response pathway, potentially leading to the identification of novel drug targets that exploit this vulnerability.

## Conclusion

**Fig. 8:**
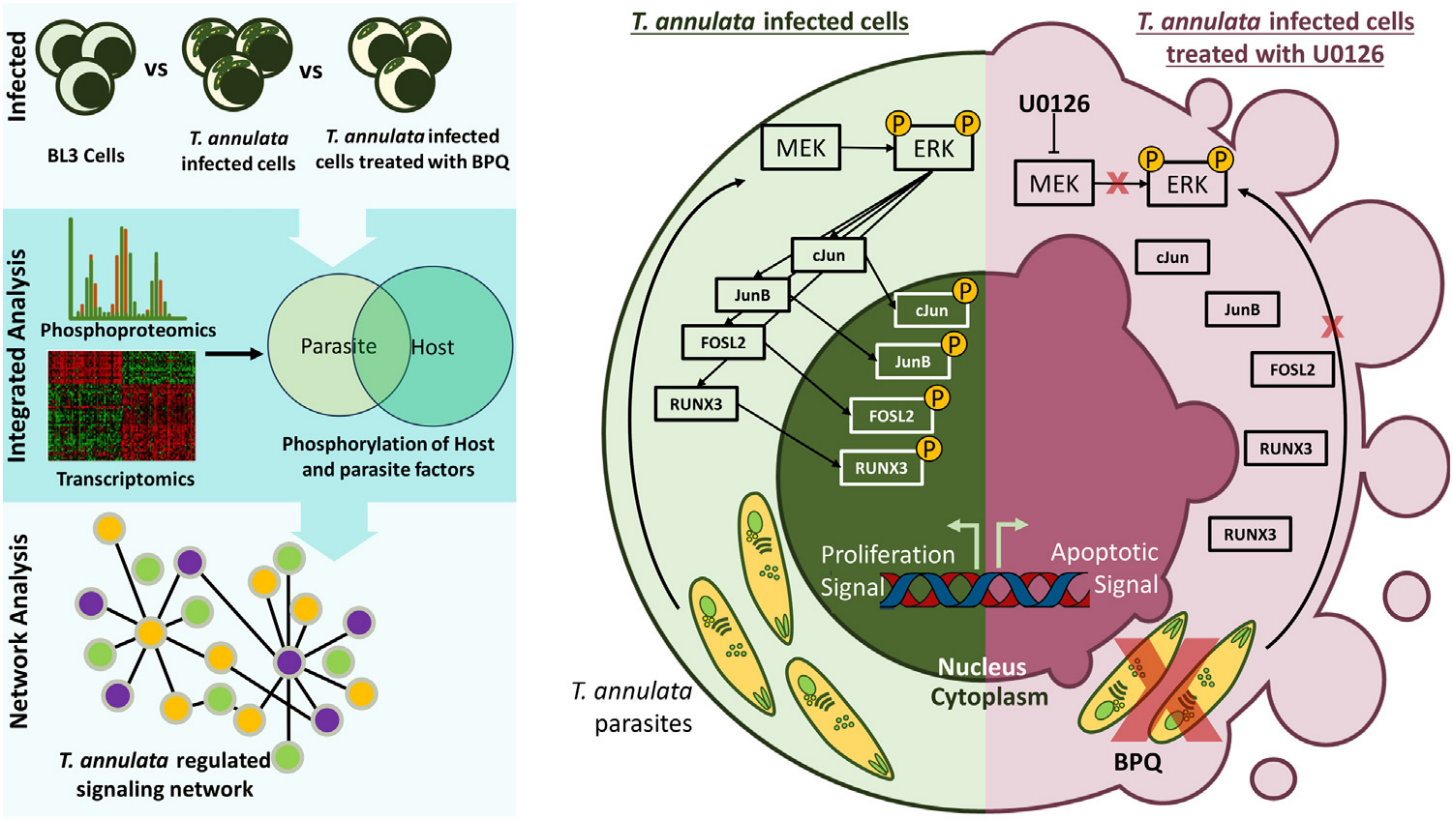
***T. annulata* manipulates host phosphosignaling in infected leukocytes.**

Our study is among the first to integrate phosphoproteomics with transcriptomics, unveiling a comprehensive map of *T. annulata*–induced phosphosignaling within infected cells. This innovative approach significantly advances our understanding of phosphorylation's critical role during parasite infection, extending far beyond the mere identification of parasite phosphoproteins achieved in prior studies. This investigation led to the discovery of unexpected regulatory pathways, including a prosurvival ERK-dependent network involving key transcription factors (Fig. 8). This finding underscores the remarkable power of phosphoproteomics in dissecting intricate cellular signaling mechanisms. Furthermore, our analysis revealed parasite-mediated manipulation of host protein phosphorylation and the identification of novel phosphorylation sites within *Theileria* proteins themselves (p104, TA14055, Hap complex factors). When integrated with the infected cell phenotype data, these comprehensive phosphoproteomic resources provide a valuable foundation for elucidating the mechanisms by which the parasite promotes host cell transformation. This knowledge has the potential to significantly accelerate therapeutic development efforts. We posit that our datasets will serve as a foundation for further validation and mechanistic studies of novel proteins implicated in *T. annulata's* manipulation of host cell signaling pathways.

## Data availability

RNA sequencing (RNAseq) data generated in this study have been deposited in the National Center for Biotechnology Information Sequence Read Archive (SRA) [https://www.ncbi.nlm.nih.gov/geo/], accessible through SRA Series accession number SUB14655643 (PRJNA1147908). Mass spectrometry proteomics data are available through the MassIVE Consortium using the MassIVE Dataset Submission (1.3.2) workflow with the dataset identifier MSV000094978. The annotated spectra file for the data can be accessed using the search key(BL3 vs TA - 4bnp1gjnfv, TA vs TA_BPQ(host) - yfqnv6s2wl and TA vs TA_BPQ(parasite) - fcfvvxzrk9) on the MS Viewer website (https://msviewer.ucsf.edu/prospector/cgi-bin/msform.cgi?form=msviewer).

## Supplemental data

This article contains supplemental data.

## Conflicts of interests

The authors declare no competing interests.

## Figures and Tables

**Supplemental Figure 5:**